# Evaluation of Serum Interleukins-6, 8 and 10 Levels as Diagnostic Markers of Neonatal Infection and Possibility of Mortality

**Published:** 2013-12

**Authors:** Hassan Boskabadi, Gholamali Maamouri, Jalil Tavakol Afshari, Shahin Mafinejad, Golkoo Hosseini, Hesam Mostafavi-Toroghi, HamidReza Saber, Majid Ghayour-Mobarhan, Gordon Ferns

**Affiliations:** 1 Department of Pediatrics, Ghaem Hospital, School of Medicine, Mashhad University of Medical Sciences, Mashhad, Iran; 2 Immunology Research Center, Avicenna Research Institute. Mashhad University of Medical Sciences, Mashhad, Iran; 3 Biochemistry of Nutrition Research Center, School of Medicine, Mashhad University of Medical Sciences, Mashhad, Iran; 4 Brighton & Sussex Medical School, Falmer, Brighton BN1 9PH, United Kingdom

**Keywords:** Infection, Interleukin-6, Interleukin-8, Interleukin-10, Newborn, Sepsis

## Abstract

***Objective(s):*** Bacterial infection contributes substantially to neonatal morbidity and mortality. Early diagnosis of neonatal sepsis is difficult because clinical signs are non-specific. We have evaluated serum IL-6, 8 and 10 as potential early diagnostic markers of neonatal infection and their relationship to mortality rate and poor prognosis.

***Materials and Methods*** *: *A total of 84 infants, aged ≥ 72 hr were enrolled in this prospective case-control trial. The case group (n=41) included babies with clinical and laboratory findings compatible with sepsis and/or positive blood or cerebrospinal fluid cultures. The control group (n=43) included healthy infants. IL-6, 8 and 10 were measured for all infants. Receiver-operating characteristic (ROC) curves were used for the determination of thresholds.

***Results*** *:* Statistically significant differences were observed between control and case groups for serum median level of IL-6, 8 and 10 (*P*<0.001). IL-6 cut-off values of 10.85 Pg/ml for discriminating between cases and controls and 78.2 Pg/ml for predicting mortality are suggested. IL-8 at a cut-off value of 60.05 Pg/ml was valuable for differentiation of definite versus indefinite infection.

***Conclusion*** *:* Evaluating the IL-6, 8 and 10 simultaneously, could improve the sensitivity and specificity of early diagnosis of the neonatal sepsis. Regarding our results, interleukin 6 had the greatest value for predicting infection and possible mortality, whereas IL-8 was valuable for diagnosing definitive infection.

## Introduction

Neonatal sepsis is a life threatening condition that contributes significantly to morbidity and mortality in newborn infants (1). Early diagnosis of neonatal bacterial infections is difficult because clinical signs are often non‐specific, variable and may initially be subtle. Early definitive diagnostic tests are not available (-). Isolation of microorganisms from body fluids such as blood, cerebrospinal fluid (CSF) and urine remains the gold standard method for diagnosis of neonatal infection, but microbiological culture is not available before at least 36-48 hr (5, 6). It is recommended that neonates who develop signs of sepsis should start empirical antimicrobial therapy. Therefore, many infants without sepsis usually receive at least 2-3 days of antibiotic therapy (7). This problem causes prolonged hospitalization and a considerable economic burden, particularly in developing countries with poorly-equipped Neonatal Intensive Care Units (NICUs). Recently various hematological and biochemical markers, for example immature/total neutrophil ratio, nucleated red blood cell, C‐reactive protein (CRP), neutrophil CD64 and cytokines have been evaluated as potential indicators for early identification of septic infants (8, 9). Cytokines are promising diagnostic markers and their levels are increased early in the infectious process (7, 10). Interleukins are pro-inflammatory cytokines predominantly produced by monocytes, activated macrophages and endothelial cells. Recent studies have reported serum interleukins are altered early in the course of neonatal bacterial infections (11). Finding a reliable laboratory test as a marker for immediate detection of infection with acceptable sensitivity and specificity has always been controversial among investigators. In previous reports IL-6, 8 and 10 were not evaluated simultaneously. In the current study, we sought to evaluate the serum levels of IL-6, 8 and 10 as early diagnostic markers in late neonatal infection (definitive infection and clinical sepsis) and their relationship to clinical outcomes. We also aimed to determine the specificity and sensitivity of interleukins (alone or in combination) in early detection of late neonatal infection, and suggest cut-off values for studied interleukins in order to detect infections and predict poor prognosis neonatal sepsis. 

## Materials and Methods

This was a prospective case-control study conducted between 2006 and 2010, at the Neonatal Intensive Care Unit (NICU) and Emergency Department in Ghaem Hospital, Mashhad, Iran. Eighty four neonates aged 72 hr or more were eligible for the study and were all enrolled. The Ethics Committee of Mashhad University of Medical Sciences (MUMS) approved this study and all parents signed informed consent.

Neonates were categorized into two groups: cases and controls, on the basis of their clinical and laboratory manifestations. The case group (n=41) was divided into definitive infection and indefinite infection (clinical sepsis) subgroups. The definitive infection subgroup (n=22) was defined as having clinical and laboratory findings compatible with sepsis and positive blood or CSF cultures. The clinical sepsis subgroup included neonates with clinical and laboratory evidence of infection, and a negative blood and CSF culture. Clinical signs and symptoms of sepsis consisted of respiratory problems (tachypnea, grunting, intercostal retractions, apnea and need for ventilation), gastrointestinal problems (feeding intolerance and abdominal distension), neurological manifestations (seizure and irritability), cardiovascular and blood pressure instability and general signs (fever, lethargy). Laboratory findings included leukocytosis (WBC>20000/µl), leukopenia (WBC<5000/µl), thrombocytopenia (Plt<150000/µl) and positive C-reactive protein (CRP>6 mg/dl). Neonates with at least two clinical signs and one positive laboratory finding were classified to the clinical sepsis subgroup (n=19). Exclusion criteria were congenital malformations, congenital infections associated with TORCH and not enough blood sampling. The control group (n=43) included healthy infants who had been referred to the clinic for thyroid screening test or physiologic hyperbilirubinemia at 3-5 days of life. 

Complete data (birth weight, weight on admission, age, sex, gestational age, APGAR score and clinical signs), maternal data (age, complications related with pregnancy and labor, mode of delivery and parity) and risk factors for infection and laboratory values were collected and recorded for all neonates by a neonatal research fellow. 

Before administration of antibiotics by venipuncture, two milliliters of blood was taken during sepsis screening from neonates who were recruited to the study for IL-6, 8 and 10 determination and other laboratory tests. IL-6, -8, -10 enzyme‐linked immunosorbent assay (ELISA) kits (Bender Med system GmbH) were used to estimate the serum level of IL‐6, 8 and 10, and the collected data was analyzed by the logistic regression method. All samples were assayed in duplicate. Blood culture, cerebrospinal fluid culture and urine culture were also performed.

Descriptive and analytic tests (Mann-Whitney test, the Student t-test and chi-square test) were performed using Statistical Package for Social Sciences (SPSS) software as appropriate. Statistical comparison between the groups (definitive infection group, clinical sepsis group and control group) was performed using one-way Analysis of Variance (ANOVA) and *post hoc* tests were used to determine the significance of difference between 3 groups. Sensitivity and specificity were calculated for IL-6, 8 and 10. Receiver-operating characteristic (ROC) curves were used for the determination of thresholds for the sepsis group versus healthy neonate group. *P*-value of <0.05 was considered statistically significant.

## Results

Complete data was obtained for 84 infants who were categorized into case (n=41) and control (n=43) groups respectively. No significant differences (*P*>0.05) were found between cases and controls in gender, birth weight, gestational age, age on admission and APGAR score (Table 1). Regarding the short half-life of serum interleukins, the effect of delivery factors and asphyxia was negligible due to participation of babies aged 3 days and older.

**Table 1:** Newborn characteristics among subgroups

	Case group		Control group	*P*-value*
	Definitive infection	Clinical sepsis		
Number of participants	22	19	43	0.721
Gestational age (weeks)±SD^a^	35.6±3.76	35.89±4.01	36.12±3.38	0.785
Birth weight (g)±SD	2250±701	2230±730	2212±680	0.843
Age (day)±SD	9.84±6.48	9.89±6.07	8.93±7	0.173
APGAR score (5 min)±SD	8.4±1.5	8.5±1.24	8.62	0.567

Data is presented as mean (SD);

a Standard Deviation;

* *P*-value of comparing case and control groups by t-test

Among the definitive infection subgroup, positive blood cultures were reported in 20 cases; five as Gram negative bacilli, seven as *Klebsiella pneumonia*, six as *Escherichia coli*, and two as *staphylococcus* *epidermis.* CSF culture was positive in four infants. The mortality rate was approximately 17% for the case group. 

Significant differences were observed between control and case groups in median serum IL-6, 8 and 10 concentrations (Table 2).

The median serum IL-6 levels of case and control groups were 105.9 Pg/ml (32.5-259.5 Pg/ml, CI 95%) and 4.5 Pg/ml (2.15-7.0 Pg/ml, CI 95%), respectively (Table 2). 

**Figure 1:**
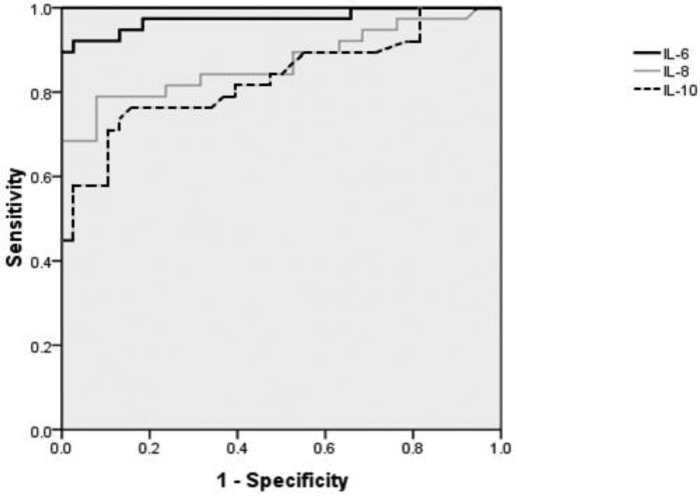
ROC curves indicating sensitivity and specificity cut-offs of IL-6, 8 and 10 for predicting case and control subjects. IL-6 at a cut off value of 10.85 Pg/ml and IL-8 at a cut off value of 64.05 Pg/ml

Serum IL-6 at a cut-off value of 10.85 Pg/ml had a sensitivity of 92.5%, specificity of 96.6%, positive predictive value (PPV) of 97%, negative predictive value (NPV) of %93 for discriminating cases from controls (Table 3 and Figure 1). The median IL-6 level of surviving newborns versus non-surviving cases was 8.3 (Pg/ml) vs. 339.4 (Pg/ml) (*P*<0.001). For predicting mortality, serum IL-6 at a cut-off value of 78.2 Pg/ml had sensitivity, specificity, PPV and NPV of 85%, 76%, 25% and 98%, respectively (Table 3).

ROC: Receiver-operating characteristic

IL: Interleukin

The median serum levels of IL-8 for neonates with definite infection (positive culture) versus neonates without definite infection were 440.7 Pg/ml (268.1-636.1 Pg/ml, CI 95%) vs. 21.1 Pg/ml (12-281 Pg/ml, CI 95%), respectively (*P*<0.001). IL-8 at a cut-off value of 60.05 Pg/ml was valuable for differentiation of definite versus indefinite infection. Sensitivity, specificity, PPV and NPV for IL-8 were 93.7%, 65%, 44%, 97%, respectively, at this cut-off (Table 3 and Figure 1).

The median serum level of IL-10 was found to be significantly higher among the case group compared with the control group (66.5 vs. 6.0 Pg/ml; *P*< 0.001) (Table 2). In addition, by evaluation and considering all 3 ILs in combination among newborns, sensitivity and specificity for discrimination between case and control groups were found to be 89% and 100% respectively.

## Discussion

The results of our study indicated that the levels of all measured interleukins were higher in neonates with sepsis compared with healthy neonates. Also serum IL-6 was higher among neonates who died versus survivors. Median serum concentrations of ILs showed an increasing pattern from control to clinical sepsis and definite infection groups, respectively.

Almost 10 percent of newborns are evaluated for infection within the first month of life and more than 90% of them receive antibiotics (12) that may be unnecessary. A reliable method for ruling out sepsis would be useful in the clinical setting as antibiotics could be withheld (11, 13). 

***The role of IL-6 for early diagnosis of neonatal infection***

IL-6 is a sensitive marker in the early phase of infection. However it cannot be used as a reliable marker in the later stages of disease, due to its short half-life (7, 11, 14). The current study shows that the median serum level of IL-6 is higher in cases with sepsis in comparison with control healthy infants. Previous studies have shown that newborns display higher percentages of IL-6 and IL-8 than adults (15). There is a sharp rise in IL-6 concentration following exposure to bacterial products (16). IL-6 has also the highest sensitivity (89%) and negative predictive value (91%) at the onset of infection compared with other biochemical markers, including CRP, TNF, and E-selectin (17). Consistent with our findings, Maamouri *et al* Reported a higher mean serum level of IL-6 in clinical sepsis compared with control group (185 vs. 5 Pg/ml) (18). In a study by Krueger *et al*, infants were classified into documented infection, possible infection and healthy groups and mean serum IL-6 levels were found to be 1920, 50 and 8 Pg/ml, respectively (19). In another study by Romagnoli *et al*, higher serum IL-6 and 10 levels were associated with neonatal sepsis (20).

**Table 2:** Median level of IL^a^-6, 8 and 10 in the case and the control groups

Marker	Case group Median (interquartile range)	Control group Median (interquartile range)	*P*-Value
IL-8 (Pg/ml)	335.7(69.8-614.7)	18.3(8-21.1)	<0.001
IL-6 (Pg/ml)	105.9(32.5-251.5)	4.5(2.1-7)	<0.001
IL-10 (Pg/ml)	66.5(10.3-200)	6.0(4.2-8.5)	<0.001

IL: Interleukin

Values are expressed as mean±SD (for normally distributed data) or median and interquartile range (for non-normally distributed data).

Comparison between the case and the control groups were made using paired t-test or Wilcoxon test for normally and non-normally distributed data, respectively

***Association between serum level of IL-8 and neonatal sepsis***

In this study serum IL-8 was demonstrated to be more efficient in the diagnosis of definite infection in comparison with IL-6 and 10. Increasing the cut off for serum IL-8 to more than 64 Pg/ml, predicted definite infection with sensitivity and specificity of 94% and 65%, respectively. Edgar *et al* demonstrated significant rises of IL-6 and 8 among cases with positive blood culture (21). Other investigators have reported elevated serum IL-8 levels in early and late neonatal sepsis to have a sensitivity and specificity of 80-91% and 76-100%, respectively (22). Boskabadi *et al* reported IL-8 concentration to be 3.3 times higher in mortal cases compared with surviving ones (23). In a study by Franz *et al*, combination of IL-8 and CRP showed a sensitivity and specificity of 91% and 73%, respectively, for early diagnosis of neonatal sepsis. They have also reported that premature infants who succumbed due to a culture proven sepsis had extremely high levels of circulatory IL-8, greater than 10000 Pg/ml (24). Chemokines and pro-inflammatory cytokines are essential for host defense against microbial infection but excessive influx of activated pro-inflammatory mediators can contribute to deleterious results, leading to widespread small-vessel damage, multi-organ dysfunction and death (25).

***Association between serum level of IL-10 and neonatal sepsis***

Boskabadi *et al* have reported that quantitative measurements of IL-10 could assist neonatologists in predicting the severity of infection. They concluded that IL-10 at a cut off value of 14 Pg/ml is a valuable tool for this purpose (26). Although mean IL-10 concentration was higher in our case group, threshold values defined by the ROC curve were not as valuable as IL-6 and 8 for prediction and diagnosis of neonatal infection.

**It seems that measurement of serum IL-6, 8, and 10 in newborns in whom there is a suspicion of sepsis, using the cut-off values reported in this study may predict neonatal sepsis and prevent overt hospitalization and antibiotics prescription. The results showed that among these markers, interleukin 6 had the greatest predictive value for determining ill newborns (case group) and predicting mortality rate, while IL-8 was more valuable for diagnosing definitive infection.**

**Table 3:** Sensitivity, specificity, positive predictive value and negative predictive value of interleukins

Diagnostic test and cut-off value	Sensitivity (%)	Specificity (%)	Positive predictive value (%)	Negative predictive value (%)
IL^a^-6 >10.85 (Pg/ ml) Case vs. control	92.5	97.6	97.3	93.1
IL-6 >78.2 (Pg/ ml) Mortality vs. alive	85	76	25	98
IL-8 >64.05 (Pg/ ml) Definitive sepsis vs. indefinite	93.7	65	44	97

a *IL: Interleukin *

We observed a sensitivity of 89% and specificity of 100% considering all 3 ILs in combination for identifying infected neonates, indicating that sensitivity and specificity can significantly be improved by using cut off values for IL-6, 8 and 10 together. Further studies with large sample size in subgroups of suspected infective infants and comparison of sensitivity and specificity of these markers in various cut-off values with traditional markers such as CRP and neutrophil CD64 are encouraged. 

In this study we had limitations such as small sample size. Also evaluation of Group B Streptococcus on mothers was not possible due to lack of facilities.

## Conclusion

A significant increase in serum levels of IL-6, 8 and 10 provides a valuable marker for early diagnosis of sepsis and predicting outcome. IL-6 appears to be a better marker of sepsis and mortality in comparison with IL-8 and 10. Serum level of IL-8 is more associated with definite infection (positive cultures) compared with the other interleukins studied.
